# Rethinking Pneumococcal Urinary Antigen Testing in the Emergency Department: From Reflex Testing to Reasoned Use

**DOI:** 10.7759/cureus.104292

**Published:** 2026-02-26

**Authors:** So Sakamoto, Naoya Itoh

**Affiliations:** 1 Department of Emergency Medicine, Asahi General Hospital, Asahi, JPN; 2 Department of Infectious Diseases, Nagoya City University, Nagoya, JPN

**Keywords:** antimicrobial stewardship, community-acquired pneumonia (cap), emergency department, gram stain, pneumococcal pneumonia, pneumococcal urinary antigen

## Abstract

Pneumococcal urinary antigen testing (PUAT) is often ordered reflexively in the emergency department (ED) because it is rapid and noninvasive. However, the ED-relevant question is not whether PUAT can detect pneumococcal antigen, but whether it reliably changes what clinicians do next: initial antibiotic selection and/or early narrowing, diagnostic strategy, disposition, and ultimately patient outcomes. The accuracy of PUAT depends on the population being tested; in cohorts enriched for confirmed pneumococcal pneumonia, performance can be overestimated. In the ED, a negative PUAT cannot reliably rule out pneumococcal disease. In addition, PUAT cannot identify polymicrobial infection, and positive results can be misinterpreted without attention to clinical context (e.g., recent infection, polysaccharide vaccination, and carriage biology). Consequently, routine PUAT may become a low-yield ritual with limited decision impact. We propose redesigning PUAT around actionability: order PUAT only when a positive result would plausibly support narrowing antibiotics in a clinically appropriate context (low aspiration and limited concern for polymicrobial infection) and when reliable sputum information is unavailable. A simple "3C" framework can help shift PUAT from reflex testing to reasoned use in ED practice.

## Editorial

Community-acquired pneumonia (CAP) is one of the most common diagnoses in the emergency department (ED) [1]. In a setting defined by time pressure, diagnostic uncertainty, and frequent interruptions, tests can quietly drift from decision support into reflex. Pneumococcal urinary antigen testing (PUAT) can be perceived as an "easy" microbiologic add-on in ED workflows, including in Japanese clinical practice, because it is rapid and noninvasive and is often described as highly specific; as a result, it can drift into reflex testing unless linked to a clear decision target [2,3]. However, the ED question is not whether PUAT can detect pneumococcal antigen, but whether routine PUAT changes what clinicians actually do: antibiotic selection, de-escalation, diagnostic strategy, disposition, and ultimately patient outcomes. Here, "de-escalation" includes both (1) immediate narrowing of initial empiric therapy when a rapid positive result makes a narrower regimen reasonable and safe and (2) early narrowing after admission once early trajectory and additional microbiology are reviewed (often within 24-48 hours).

Guidelines and reviews describe practical advantages of PUAT, including ease of sampling and rapid turnaround, and note that urinary antigen detection may be less affected by prior antibiotics than cultures in some contexts [1,3]. These properties make PUAT tempting to add "by routine," particularly when ED teams feel pressured to provide microbiologic answers early. But "easy" is not the same as "useful." A test can become reflexive when it is ordered without a pre-test question and when its result does not reliably change management [4]. In many EDs, empiric CAP therapy for hospitalized patients is protocolized, and antibiotic decisions are driven primarily by illness severity, aspiration risk, and concern for drug-resistant pathogens rather than by a single rapid test result [1]. If the result rarely changes actions, routine testing risks becoming a diagnostic ritual.

That said, there are ED scenarios where PUAT can be genuinely useful. The highest-yield use case is when clinicians are prepared to act on a positive result, specifically, to consider narrowing therapy toward pneumococcus-targeted agents (e.g., penicillin/aminopenicillin) in a patient with low aspiration risk and limited concern for polymicrobial infection, particularly when sputum information is unavailable or unreliable due to poor specimen quality or prior antibiotic exposure (a decision-directed proposal) [1,3].

A central pitfall is to generalize PUAT accuracy estimates derived from cohorts enriched for "confirmed pneumococcal pneumonia" (often including bacteremic disease) to the undifferentiated ED population of "pneumonia suspected." Diagnostic test performance, including sensitivity, specificity, and predictive values, is sensitive to the study denominator and reference standard; recent laboratory utilization guidance explicitly emphasizes that apparent accuracy can shift depending on the population tested and the imperfect nature of microbiologic gold standards [5]. When PUAT is applied to the broad ED population, many results will be negative (without safely excluding pneumococcus), and a nontrivial number will be positive but still require context to avoid overinterpretation [3,6,7].

This context dependence is well illustrated by the systematic review and meta-analysis by Yasuo et al., which evaluated urinary antigen testing among adults with acute respiratory failure in whom pneumonia was suspected (30 studies; n=12,366; pneumococcal pneumonia prevalence 12.5%). Pooled sensitivity was 0.66 and specificity 0.90, supporting a rule-in role but a weak rule-out role [6]. The authors also showed that estimates varied depending on the composite reference standard used, reinforcing that "accuracy" is not absolute but definition-dependent [6]. In practical ED terms, a negative PUAT should not be used to exclude pneumococcal disease, and a positive PUAT still requires interpretation within the full clinical picture.

Even if PUAT is treated primarily as a rule-in test, the next pragmatic question is yield. In a Korean propensity score-matched cohort of 1,257 hospitalized community-onset pneumonia patients, PUAT positivity was 13%, with an overall sensitivity of 56.5% and a specificity 90.9% for pneumococcal pneumonia (definitive + probable definitions) [7]. Similarly, in a large Danish multicenter cohort of 2,449 immunocompetent adults hospitalized with CAP, urinary antigen testing (UAT) was performed in only 26.7% within 48 hours; PUAT was positive in 8% of those tested [8]. Low positivity rates mean that routine ED testing across broad "CAP-suspected" populations will inevitably generate many results that do not meaningfully refine management.

Low yield also carries a cost. For example, a commonly used PUAT kit has been cited at approximately $17 per test (US Centers for Medicare & Medicaid Services (CMS) pricing), translating to roughly $425 per positive result in low-yield settings [3]. For an ED trying to prioritize high-value care, routine PUAT without a clear decision target risks consuming resources without creating commensurate clinical value.

Another limitation is structural: PUAT cannot identify polymicrobial infection. This matters because CAP, especially in older adults, often reflects overlapping processes (viral-bacterial coinfection, aspiration-associated flora, and mixed typical pathogens) [1,3]. PUAT may detect pneumococcal antigen but cannot indicate whether another organism is also clinically important. In the Korean cohort, 22.1% of PUAT-positive patients had another pathogen detected by conventional methods, suggesting true mixed infection and/or diagnostic overlap [7]. If clinicians treat a positive PUAT as proof of monomicrobial pneumonia and narrow therapy too aggressively, clinically relevant copathogens (e.g., *Haemophilus influenzae*) or aspiration-associated anaerobes may be undertreated.

A positive PUAT should not be treated as proof of monomicrobial pneumonia. In real-world cohorts, additional pathogens are not uncommon even when PUAT is positive, and narrowing decisions are therefore best guided by aspiration risk, illness severity, supporting microbiology, and local epidemiology [1,7]. Importantly, PUAT does not exclude atypical pathogens; when atypical coverage is indicated based on severity or epidemiologic risk, it should not be discontinued solely on the basis of a positive PUAT [1,3].

This is where sputum Gram stain can add a different kind of value, not because it is universally "more accurate" than PUAT, but because it provides different information. Meta-analyses suggest that, in high-quality sputum specimens, Gram stain specificity is high (e.g., 0.87-0.91 for *S. pneumoniae*; 0.96-0.97 for *H. influenzae*), while sensitivity is moderate (0.59-0.69 for pneumococcus) [9,10]. Gram stain is limited by specimen quality and operator dependence, and current evidence does not support blanket claims that Gram stain is "superior to PUAT" for etiologic diagnosis [9,10]. However, Gram stain uniquely provides visual information (morphotype, relative abundance, specimen quality, and polymicrobial patterns) that PUAT cannot deliver. In an ED where aspiration risk and mixed infection are common, "seeing the sputum" can strengthen clinical reasoning when feasible, especially when narrowing antibiotics is being considered (operational perspective) [9,10].

Interpretation of PUAT also requires a time axis. The Japanese Respiratory Society (JRS) guideline explicitly cautions that pneumococcal urinary antigen can remain positive for months after infection and may mislead clinicians when patients present with recurrent pneumonia [2]. The same guideline notes potential false positivity due to shared antigens with other streptococci (e.g., *Streptococcus mitis*) and emphasizes cautious interpretation in context [2]. Reviews also describe transient positivity after pneumococcal polysaccharide vaccination, making vaccination history relevant to interpretation [3]. Carriage biology further complicates interpretation: in healthy colonized children, pneumococcal urinary antigen can be positive and declines with age, illustrating how antigen detection can occur even in the absence of invasive disease [11]. These observations strengthen the argument against reflex ED ordering: routine testing increases the chance that results will be interpreted without attention to recent infection, vaccination context, or exposure history.

Guidance often suggests restricting PUAT to severe CAP [1]. Yet severe CAP in the ED typically triggers broad empiric therapy by default, and PUAT rarely changes day-0 antibiotic selection in that context. If the intended benefit of PUAT is stewardship (de-escalation), the "severe-only" framing can collide with real ED decision-making: the sicker the patient, the less comfortable clinicians are in narrowing early, particularly when aspiration risk or polymicrobial infection is plausible.

A prospective observational Japanese cohort study by Ito et al. illustrates this implementation gap. Among PUAT-positive pneumococcal CAP patients, penicillin-targeted therapy initiated within 24 hours of PUAT was implemented in only 19.6% [12]. The strongest positive predictor of targeted therapy was Gram stain showing gram-positive diplococci, while aspiration pneumonia and higher CURB-65 severity were negative predictors [12]. In other words, clinicians were least likely to narrow therapy in the patients where "severe-only testing" would be emphasized, because severity and aspiration risk drive concern for mixed pathogens and justify broader coverage. Outcome analyses showed no significant difference in treatment failure or 30-day mortality between targeted and non-targeted groups [12]. This pattern suggests a practical reframing: "severe vs. non-severe" is an imperfect axis for deciding PUAT use. A more operational axis is "will this result change management in this patient's risk context?".

A key justification for routine PUAT is that it could improve outcomes by enabling earlier pathogen-directed therapy. Yet hard outcome benefits remain unproven, and real-world stewardship translation is inconsistent. In US hospitals, PUAT has been described as a missed opportunity for antimicrobial stewardship, reflecting that testing does not reliably translate into de-escalation [4]. More concerning, Kern et al. evaluated whether UAT within 48 hours impacted 30-day mortality in a multicenter cohort; it did not [8]. Moreover, UAT was associated with higher odds of broad-spectrum antibiotic use on hospital day 3 and at discharge [8]. The nuance is that among pneumococcal UAT-positive patients, broad-spectrum therapy at discharge was lower than in UAT-negative patients, suggesting that a positive result can support narrowing [8]. However, because positivity rates are low, routine testing may yield only small population-level stewardship effects, and testing alone does not guarantee better antibiotic use.

These observations point to a more actionable message for ED practice: PUAT should be redesigned around decision impact, and its timing should be intentional. If the primary intended value of PUAT is stewardship rather than immediate ED decision-making, the ED visit is not necessarily the optimal time to test. In many hospitalized patients, PUAT could be performed within the first 24 hours of admission, after cultures are obtained, severity and aspiration risk are clarified, and an early clinical trajectory is observed. This timing may reduce reflex ED ordering while preserving the potential stewardship benefit of a positive result. A test can be rapid and still not be urgent.

A brief pre-test pause can help convert reflex testing into decision support. The questions are simple: What decision will this result change (today vs. tomorrow)? Is aspiration risk or mixed infection concern high enough that narrowing would be unsafe even if PUAT is positive [12]? Is sputum available, and could a Gram stain inform polymicrobial risk or a dominant pathogen pattern [9,10]? Is there recent pneumococcal pneumonia, recent vaccination, or an exposure context where antigen positivity could mislead interpretation [2,3,11]? If the answer is that PUAT will not change management, then routine ordering is difficult to justify.

To operationalize this approach, Table 1 proposes a simple ED rule: order PUAT only when it is likely to change antibiotics and when the clinical context supports interpreting a positive result as actionable rather than merely interesting.

**Table 1:** PUAT in the ED: the 3C rule (and when to avoid testing) PUAT: pneumococcal urinary antigen testing; ED: emergency department Table Credit: So Sakamoto with reference to [1-3,6-8,11,12]. Interpret positives in clinical context (recent pneumococcal infection, polysaccharide vaccination, and carriage biology, especially in children).

Order PUAT only if ALL 3 C's are met	
Change	A positive result would change antibiotics (narrowing is on the table)
Clean	Low aspiration/mixed infection concern
Clues missing	No reliable sputum clue (no sputum, poor sample, or antibiotics before sampling)
Avoid PUAT if ANY of the following applies	
Can't rule out	You need a rule-out test (a negative PUAT does not rule out pneumococcal disease)
Care won't change	You would treat the same regardless (result won't alter antibiotics)
Confounded	Aspiration/mixed infection is likely (a positive PUAT does not confirm monomicrobial pneumonia)

It is also reasonable to acknowledge one practical contrast: urinary antigen testing for *Legionella* often occupies a different category because rapid alternatives may be limited and results can more directly shift empiric therapy [1,3]. *Legionella *testing is typically recommended in specific clinical or epidemiologic contexts (e.g., severe CAP, outbreak/travel exposure, or other risk features), because timely identification can directly shift empiric therapy toward appropriate atypical coverage when alternative rapid diagnostics are limited [1,3]. In contrast, PUAT is often obtained for convenience as a broad add-on, yet it is neither a reliable rule-out test nor a guarantee of monomicrobial disease; therefore, a positive result may or may not justify narrowing depending on aspiration risk, mixed infection concern, and available microbiology [1,6-8,12]. We include this contrast to emphasize a core message of this editorial: "urine antigen testing" is not a single concept or panel to order reflexively; its value depends on the pathogen, the pretest context, and whether the result will change management.

PUAT has real strengths: speed, noninvasive sampling, and useful specificity in many settings [6]. But in ED practice, routine testing risks becoming a diagnostic ritual, producing low yield, weak rule-out information, and results that often do not change management or outcomes [6-8,12]. The evidence supports a more nuanced view: PUAT can add diagnostic yield and can support de-escalation when positive, but testing alone does not ensure stewardship benefit, and population-level outcome improvements have not been demonstrated [4,8,12]. The ED should therefore rethink PUAT as a decision tool rather than a reflex. We summarize this decision-directed approach as the ED 3C framework (Change, Clean, and Clues missing; Table 1) to shift PUAT from reflex testing to reasoned use. The most important question is not whether we can order it, but whether its result will change what we do next. Future studies should evaluate decision-directed PUAT strategies in ED workflows, linking testing to prespecified antibiotic actions and patient-centered outcomes, to better define when PUAT adds value and to inform practical, evidence-based guidance for ED use.
